# Successful Treatment of Recalcitrant Granuloma Gluteale Infantum with Topical Tacrolimus 0.03% Ointment

**DOI:** 10.1155/2021/9994067

**Published:** 2021-06-07

**Authors:** Alexander K. C. Leung, Kin Fon Leong, Joseph M. Lam

**Affiliations:** ^1^Department of Pediatrics, The University of Calgary and Pediatric Consultant The Alberta Children's Hospital, Calgary, Alberta T2M 0H5, Canada; ^2^The Pediatric Institute, Kuala Lumpur General Hospital, Kuala Lumpur, Malaysia; ^3^Department of Dermatology and Skin Sciences, University of British Columbia and Consultant Pediatric Dermatologist the BC Children's Hospital, Vancouver, British Columbia V6H 3V4, Canada

## Abstract

Granuloma gluteale infantum is a rare complication of irritant contact dermatitis. For the treatment of granuloma gluteale infantum, the diaper area should be kept clean and dry and the source of skin irritation should be removed or mitigated. For those infants who fail to respond to the conservative management, the use of topical calcineurin inhibitors should be considered. We report the successful treatment of a 12-month-old infant with recalcitrant granuloma gluteale infantum with the use of topical tacrolimus 0.03% ointment. To our knowledge, this is the second case reporting the use of topical calcineurin inhibitors in the treatment of recalcitrant granuloma gluteale infantum.

## 1. Introduction

Granuloma gluteale infantum, a rare complication of irritant diaper dermatitis, occurs most commonly during infancy [1, 2]. Clinically, the lesions present as multiple asymptomatic erythematous papules and nodules in the diaper area in the setting of primary irritant contact dermatitis [3]. The disease was originally named “vegetating bromidism” in 1891 due to its occurrence with the application of bromide-containing ointments [3]. The term “granuloma gluteale infantum” was coined in 1971 by Tappeiner and Pfleger who reported six healthy infants with a granulomatous eruption involving the gluteal region [4]. Treatment is usually conservative, consisting of eliminating sources of irritation and the use of barrier creams [1]. At times, corticosteroids have been used in the treatment of recalcitrant cases but with conflicting results [5]. Herein, we report the successful treatment of a 12-month-old infant with recalcitrant granuloma gluteale infantum with the use of topical tacrolimus 0.03% ointment. To our knowledge, this is the second case reporting the use of topical calcineurin inhibitors in the treatment of recalcitrant granuloma gluteale infantum.

## 2. Case Report

A 12-month-old boy with a 4-month history of a recalcitrant diaper rash was referred to us. The child was exclusively breastfed for 6 months at which time solid food was introduced. The child appeared well and was thriving. Stools had been loose but not watery since 6 months of age. Parents used nondisposable cloth diapers for the child. The diaper rash was treated with topical zinc oxide and multiple courses of topical antifungals, topical antimicrobials, and fluorinated steroid creams alone or in combination without much success. Past health was otherwise unremarkable. In particular, there was no history of seborrheic dermatitis, atopic dermatitis, or psoriasis. There was no history of powder preparations applied to the diaper area.

**Figure 1:**
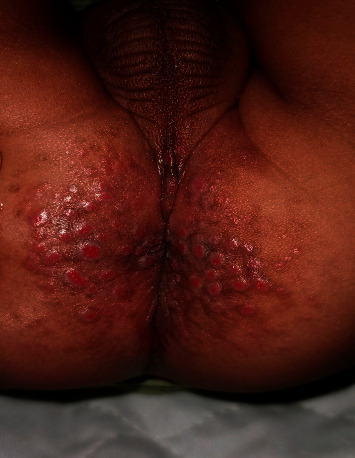
Numerous, erythematous, friable papules and nodules in the perianal and bilateral gluteal areas.

Physical examination revealed numerous, erythematous, round-to-oval, nontender papules and nodules, located on the perianal and bilateral gluteal areas (Figure 1). Some of the lesions were friable and eroded. Systemic examination was otherwise unremarkable. In particular, there was no evidence of acral and periorificial dermatitis and alopecia.

A clinical diagnosis of granuloma gluteale infantum was made. Culture from a skin swab taken from the lesion was negative for bacteria. Microscopic examination of skin scrapings in 10% potassium hydroxide was negative for fungal elements. Histologic examination of the lesions revealed acanthosis, hyperkeratosis, and spongiosis with overlying crust. Additionally, there was a dense dermal infiltrate consisting of lymphocytes, neutrophils, plasma cells, histiocytes, and scattered eosinophils. No fungal element was identified with periodic acid Schiff (PAS) stain.

**Figure 2:**
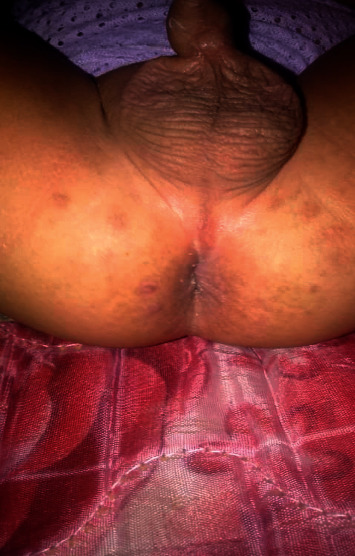
Complete resolution of the lesions 6 weeks after treatment with topical tacrolimus ointment. Postinflammatory hyperpigmentation in the form of brown macules was noted in some areas.

Parents were advised to use disposable diapers for the child and avoid irritant contact dermatitis by keeping the diaper area dry and clean with frequent diaper changes and the use of topical zinc oxide cream. The child was treated with topical tacrolimus ointment 0.03% twice a day for 6 weeks with complete resolution of the lesions leaving residual brown macules in some areas (Figure 2).

## 3. Discussion

It is estimated that 7 to 35% of infants have diaper dermatitis at any given time with a peak incidence at 9 to 12 months of age [6]. Irritant contact dermatitis is the most common type of diaper dermatitis [6]. Granuloma gluteale infantum is a rare complication of irritant contact dermatitis [1, 2]. Predisposing factors include chronic irritation with urine and feces, high-pH environment (due to breakdown of urea in the urine by fecal urease), and use of nondisposable cloth diapers, plastic pants, baby wipes, laundry detergents, starch or talc powder application, topical fluorinated corticosteroids, topical benzocaine, and bromide-containing ointments [1, 2, 5, 7–12]. Infection with *Candida albicans* has also been implicated [1, 13]. However, there is no difference in the isolation rate of *Candida albicans* from patients with granuloma gluteale infantum and from patients with other forms of dermatitis in the diaper area alone [7, 12, 14].

The average onset is between 9 and 12 months of age [3]. There is no gender predilection [3]. Clinically, granuloma gluteale infantum presents with asymptomatic, well-demarcated, firm, round-to-oval, red to purple, papules or nodules [1, 11, 14, 15]. The size of lesions can range from 0.5 to 3 cm in diameter [11, 14–16]. The surface of the lesion can be smooth, lichenified, eroded, or ulcerated depending on the stage of lesions [1]. Characteristically, the lesions occur on the convexities of the gluteal skin surfaces where there is maximum contact with the diaper and typically spare the inguinal folds [14, 15, 17] Postinflammatory hyperpigmentation and atrophic scars are potential complications, as illustrated in the present case [1, 7, 11, 16].

Histologic examination of the lesion typically shows acanthosis, parakeratosis, spongiosis, and exocytosis [1]. In addition, there is a dense perivascular dermal infiltrate with lymphocytes, neutrophils, histiocytes, plasma cells, and eosinophils [11, 14]. The histologic findings are neither specific nor diagnostic but sufficiently characteristic to exclude a number of other conditions in the differential diagnoses [14, 17].

The differential diagnosis is broad and includes Jacquet erosive diaper dermatitis, pseudoverrucous papules and nodules, Candidal diaper dermatitis, seborrheic dermatitis, atopic dermatitis, perianal streptococcal dermatitis, plaque psoriasis, bullous impetigo, juvenile xanthogranuloma, molluscum contagiosum, acrodermatitis enteropathica, nodular scabies, Langerhans cell histiocytosis, and maculopapular cutaneous mastocytosis [3, 18–22]. The distinctive features of most conditions allow a straightforward differentiation to be made (Table 1) [3, 18–22].

For the treatment of granuloma gluteale infantum, the diaper area should be kept clean and dry and the source of skin irritation should be removed or mitigated [1, 15]. The use of a barrier cream such as zinc oxide is advisable [1].

Some authors use topical corticosteroids for the treatment of granuloma gluteale infantum, especially for recalcitrant cases [23, 24]. Other authors object the use of topical corticosteroids because topical corticosteroid application may lead to an increase in the number and size of the lesions [5, 14, 15]. The use of topical corticosteroid on delicate skin in children is not without risk [25, 26]. Compared with adults, children are at higher risk of both local and systemic effects [25, 26]. Local adverse events, particularly on delicate skin areas, include skin atrophy, depigmentation, striae, perioral dermatitis, folliculitis, telangiectasia, decreased subcutaneous adipose tissue, rosacea, and steroid acne [25, 26]. Percutaneous absorption of corticosteroids may lead to systemic side effects which include growth retardation, hypothalamic-pituitary-adrenal suppression, cataracts, glaucoma, Cushing's syndrome, and osteopenia/osteoporosis [25, 26]. Given the potential role of corticosteroid application in the induction of granuloma gluteale infantum and the adverse events associated with its use, the use of topical corticosteroids in the treatment of granuloma gluteale infantum is not recommended [5, 14, 15].

Topical calcineurin inhibitors, notably tacrolimus, have emerged as a reasonable treatment option for the treatment of recalcitrant granuloma gluteale infantum with promising results. Ramos Pinheiro et al. reported an 18-month-old girl with refractory granuloma gluteale infantum unresponsive to multiple treatments including barrier creams, various topical antifungal agents, topical antibiotics, and topical corticosteroids including hydrocortisone butyrate 0.1% cream and betamethasone valerate 0.1% cream [14]. The child was treated with daily pimecrolimus 0.1% cream for one month followed by tacrolimus 0.03% ointment. After four weeks of topical tacrolimus treatment, there was a complete regression of the ulcerated lesions. After eight weeks of topical tacrolimus treatment, only transient postinflammatory hyperpigmentation remained, evolving into hypopigmented residual patches subsequently. In the present case, our patient was treated with topical tacrolimus ointment 0.03% twice a day for 6 weeks with complete resolution of the lesions. Topical calcineurin inhibitors have a favorable safety profile. Percutaneous absorption has been shown to be low, and there is no evidence of systemic toxicity. Nevertheless, the Food and Drug Administration issued a black box warning for tacrolimus and pimecrolimus in January 2006 [27]. The warning includes the concern that the long-term safety of these medications has not been established and that although no definite causal relationship has been conclusively established between topical calcineurin inhibitors and malignancy, there have been rare case reports of malignancy in patients treated with these medications. The labeling advises that topical calcineurin inhibitors should be recommended as a second-line treatment and that use in children younger than 2 years of age is not recommended [28]. A Joint Task Force of the American College of Allergy, Asthma, and Immunology and the American Academy of Allergy, Asthma, and Immunology reviewed the existing data and concluded that the data did not support the use of “black box warning” on these medications [29]. Based on the current review of the literature and clinical experience of experts in the field, long-term use of topical calcineurin inhibitors does not lead to skin atrophy, enhanced percutaneous absorption, or impaired epidermal barrier function and, therefore, is suitable for use in sensitive skin areas [30]. Furthermore, there is no evidence of immunosuppression associated with their use and that these medications are effective and safe for use in infants three months of age or above. For those infants with recalcitrant granuloma gluteale infantum who fail to respond to the conservative management, we suggest that topical tacrolimus be considered.

## 4. Conclusions

Granuloma gluteale infantum is a rare complication of irritant diaper dermatitis and has to be differentiated from other dermatoses in the diaper area. In the majority of cases, granuloma gluteale infantum responds to conservative treatment such as elimination of precipitating factors and the use of barrier creams. For recalcitrant cases that do not respond to the conservative management, we suggest that the use of topical tacrolimus be considered.

## Figures and Tables

**Table 1:** Differential diagnosis of granuloma gluteale infantum with differentiating features.

Condition	Characteristics
Jacquet erosive diaper dermatitis	Well-demarcated papules and nodules with central umbilications and punched-out ulcers in the genital or perianal areas; occurrence is usually in older patients

Pseudoverrucous papules and nodules	Erythematous or gray, erosive or verrucous papules, nodules, or plaques; histopathology shows reactive acanthosis and psoriasiform epidermal hyperplasia without significant dermal infiltrate

Candidal diaper dermatitis	Beefy red, erythematous rash with a scalloped border and a sharply demarcated margin in the diaper area; satellite papules and pustules along the margin are pathognomonic

Seborrheic dermatitis	Nonpruritic, salmon-colored or erythematous, sharply demarcated patches with yellow-white, greasy scales on the diaper area, face, and intertriginous areas; scaling and crusting of the scalp

Atopic dermatitis	History of atopic disease; pruritic eruption (papules, papulovesicles, prominent scaling, excoriations, and lichenification); indistinct border; chronically relapsing

Perianal streptococcal dermatitis	Sharply circumscribed, tender perianal erythema; may be associated with rectal discomfort, rectal itching, painful defecation, and blood-streaked stools lesions

Plaque psoriasis	Well-demarcated, annular, erythematous, round or oval, pruritic plaques with loosely adherent silvery-white micaceous scales; positive Auspitz sign; pitting of the nail

Bullous impetigo	Sharply demarcated bulla without surrounding erythema; rupture of the bulla reveals a moist erythematous base that dries to form a shiny lacquer-like appearance; a narrow rim of scale at the edge of the ruptured lesion is pathognomonic

Juvenile xanthogranuloma	Asymptomatic, well-demarcated, dome-shaped, firm, rubbery, round-to-oval papule or nodule; a pink-to-red lesion with a yellow tinge initially; over time, the lesion acquires a yellow-brown or orange hue and will often flatten overtime

Molluscum contagiosum	Discrete, smooth, firm, waxy, dome-shaped papules with characteristic central dell or umbilication

Acrodermatitis enteropathica	Acral and periorificial dermatitis; diarrhea; alopecia

Nodular scabies	Extremely pruritic, erythematous nodules that can persist even after treatment of scabies; pruritus is most intense at night

Langerhans cell histiocytosis	Seborrheic dermatitis-like eruption; erythematous/reddish-brown crusted/scaly papules/maculopapules/plaques/patches; eczematous lesions; bone lesion; anemia; thrombocytopenia; lymphadenopathy; hepatosplenomegaly

Maculopapular cutaneous mastocytosis	Pruritic, erythematous to reddish-brown macules/papules on the trunk and proximal extremities; positive Darier sign

## Data Availability

No data were used to support this study.
